# Using a model comparison approach to describe the assembly pathway for histone H1

**DOI:** 10.1371/journal.pone.0191562

**Published:** 2018-01-19

**Authors:** Carlos Contreras, Minaya Villasana, Michael J. Hendzel, Gustavo Carrero

**Affiliations:** 1 Department of Mathematical and Statistical Sciences, University of Alberta, Edmonton, Alberta, Canada; 2 Departamento de Cómputo Científico y Estadística, Universidad Simón Bolívar, Caracas, Miranda, Venezuela; 3 Department of Oncology, University of Alberta, Edmonton, Alberta, Canada; 4 Centre for Science, Faculty of Science and Technology, Athabasca University, Edmonton, Alberta, Canada; Universität Stuttgart, GERMANY

## Abstract

Histones H1 or linker histones are highly dynamic proteins that diffuse throughout the cell nucleus and associate with chromatin (DNA and associated proteins). This binding interaction of histone H1 with the chromatin is thought to regulate chromatin organization and DNA accessibility to transcription factors and has been proven to involve a kinetic process characterized by a population that associates weakly with chromatin and rapidly dissociates and another population that resides at a binding site for up to several minutes before dissociating. When considering differences between these two classes of interactions in a mathematical model for the purpose of describing and quantifying the dynamics of histone H1, it becomes apparent that there could be several assembly pathways that explain the kinetic data obtained in living cells. In this work, we model these different pathways using systems of reaction-diffusion equations and carry out a model comparison analysis using FRAP (fluorescence recovery after photobleaching) experimental data from different histone H1 variants to determine the most feasible mechanism to explain histone H1 binding to chromatin. The analysis favors four different chromatin assembly pathways for histone H1 which share common features and provide meaningful biological information on histone H1 dynamics. We show, using perturbation analysis, that the explicit consideration of high- and low-affinity associations of histone H1 with chromatin in the favored assembly pathways improves the interpretation of histone H1 experimental FRAP data. To illustrate the results, we use one of the favored models to assess the kinetic changes of histone H1 after core histone hyperacetylation, and conclude that this post-transcriptional modification does not affect significantly the transition of histone H1 from a weakly bound state to a tightly bound state.

## Introduction

The nuclear protein histone H1, or linker histone, binds to DNA as it enters and exits the nucleosome. In doing so, histone H1 contributes to the folding of chromatin [1] and, *in vitro*, drives the formation of a structure formed by compact nucleosome arrays, known as the 30 nm chromatin fiber. There are multiple histone H1 subtypes expressed simultaneously in most tissues. Knockout experiments have revealed that while general depletion of H1 histones through the knockout of at least three subtypes in mouse results in altered chromatin structure and a decreased spacing between individual nucleaosomes and reduced chromatin compaction [2, 3]. Knockdown experiments in *Drosophila* have revealed an essential role in chromosome architecture and development [4]. Similarly, knockout experiments of all histone H1 subtypes in chicken cells resulted in global changes in gene regulation [5], although most genes were repressed, possibly as a result of the increased nucleosome density in these cells.

Several years ago, it was noticed that histone H1 is transiently associated with chromatin in living cells [6–8]. Using fluorescence recovery after photobleaching, it has been established that there are at least three-population of each histone H1 subtype: a freely diffusing population, a weakly bound population with residence times on the scale of seconds, and a smaller and more stably bound population with residence times on the scale of minutes. Despite the identification of these populations with different binding affinities, the dynamic association of histone H1 with chromatin has been studied and quantified using a two-population system of reaction-diffusion equations, where only a population that diffuses freely within the cell nucleus and a population that is chromatin bound are considered explicitly [9, 10]. Since such a model does not provide an explicit distinction between low and high affinities, the parameter estimates, such as the proportions of bound and free populations, obtained from fitting the model to data, have required further interpretation. In particular, the high affinity has been described explicitly simply by the bound population, and the low affinity population, not considered explicitly, has been described implicitly within the freely diffusing population [11, 12]. This implicit consideration of the low affinity state is justified by perturbation analysis arguments that capture the idea of a very rapid interaction (low affinity) in comparison to a very slow interaction (high affinity) with the chromatin [11–14].

**Fig 1:**
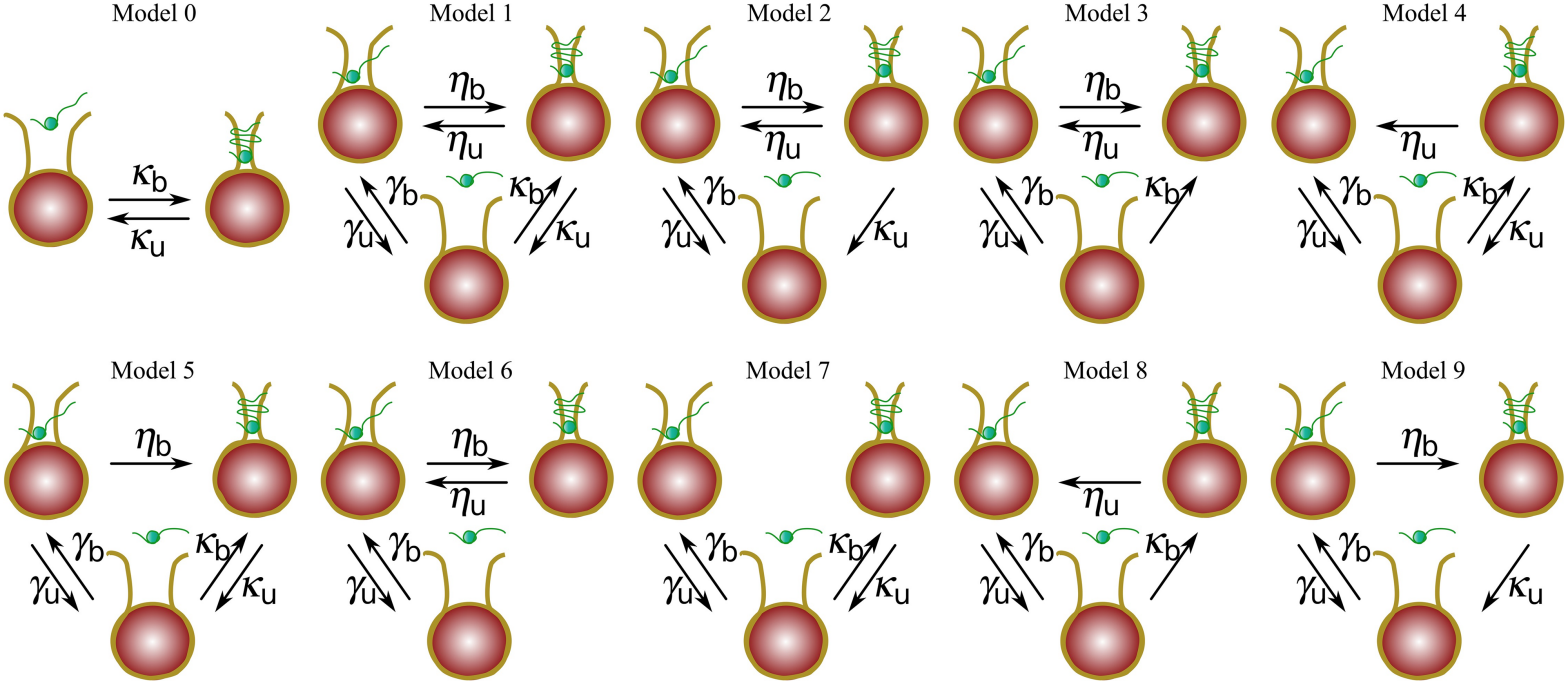
Possible assembly pathways for histone H1 with chromatin. In each diagram, histone H1 (represented by a green small ball) binds to the chromatin (DNA is represented with a yellow strip and the histone octamer, consisting of four pairs of histone proteins, with a big red ball) in either weak or strong forms. Model 0 describes a two population model consisting of a freely diffusing (left node) and bound population (right node) and a corresponding exchange rate between them. The three population models 1 through 9 consist of a freely diffusing population (lower node), and weakly (upper left node) and strongly bound (upper right node) populations; the exchange rates between the free to diffuse and weakly bound populations, free to diffuse and strongly bound populations and weakly bound and strongly bound populations are denoted by the letters *γ*, *κ*, and *η*, respectively.

Some models for the assembly pathway for histone H1 with chromatin that consider low and high affinities have been proposed and justified on a biological basis [6, 15]. When considering these low and high affinities, it becomes apparent that there are different pathways for linker histone binding to chromatin (Fig 1). This leaves us with the interesting task not only of having to translate this into a mathematical model that considers both high and low affinities explicitly, but also of differentiating among the possible assembly pathways and selecting the most appropriate one for the purpose of extracting biophysical information and understanding the association-dissociation kinetics of histone H1. Thus, the main objective of this study is to translate these feasible assembly pathways into mathematical models and perform a model comparison analysis using FRAP (Fluorescence Recovery After Photobleaching) experimental data for the purpose of selecting the most biologically feasible pathway that accounts for low and high binding affinities of histone H1. The results will be used to assess the effect of a postranslational modification, core histone hyperacetylation, on the dynamics of histone H1.

During a FRAP experiment, the population of the macromolecule under study is tagged with a fluorescent protein for its visualization. A high intensity laser source photobleaches the fluorescent protein, rendering it nonfluorescent, within a certain area of the cell. A confocal microscope is then used to collect timelapse images, which enable the rate of repopulation of fluorescent biomolecules in the photobleached area to be assessed. Thus, FRAP data consists of the measurements of the fluorescence intensity in the photobleached area over time [8, 11, 12, 16–19].

In this study, we will use FRAP data to quantify the mobility and reaction properties of histone H1 and perform a model comparison analysis to describe the association and dissociation of histone H1 with chromatin. In particular, we will consider different models that correspond to the different assembly pathways to account for the high and low affinities and compare their ability to explain the FRAP experimental data. These models comprise three interacting populations of histone H1, namely a weakly bound population, accounting for low affinity binding to the chromatin, a strongly bound population, accounting for the high affinity binding to the chromatin, and an unbound population free to diffuse. The different possible interactions among these populations will describe the different assembly pathways and therefore, models to be considered in the comparison analysis. In order to select a model that better describes the assembly pathway for histone H1 with chromatin, we will assess the fitness of the models using nonlinear least squares and carry out a model comparison and inference analysis.

Notice that when adding a second bound population to the former model that considers only a freely diffusing population and one bound population (model 0 in Fig 1), several different assembly pathways can be considered simply by accounting for the presence or absence of transition rates between any two populations. Since there is enough evidence for the existence of a rapid interaction between histone H1 and chromatin [8, 20], all the models to be considered herein contain rapid exchange rates (*γ*_*b*_ and *γ*_*u*_ in models 1 through 9 in Fig 1).

Note that the inverses $\gamma_{b}^{-1}$ and $\gamma_{u}^{-1}$, denoting the average wandering time before binding weakly and the average transition time from the weakly bound state to the freely diffusing state, respectively, are expected to be smaller than the rest of the average times that histone H1 takes to go from one state to another.

It is clear that all the models in Fig 1 describe a particular assembly pathway of histone H1 with the chromatin. For instance, in the pathways described by models 2, 6, and 9, the strong binding is possible only through a temporary transition from a weakly bound state, whereas this transition is not necessary in the other models; this is clearly seen in the pathway described by model 7 where the weak and strong binding events are independent processes.

Another aspect that can be characterized in the dynamics of the models described in Fig 1 is the dissociation pathway of histone H1 to a freely diffusing state; for example, in models 3, 6, and 8 linker histones are required to be in a weakly bound state before it can dissociate to a freely diffusing state, whereas this is not the case for the other models where histone H1 can proceed directly to a freely diffusing state without an intermediate weakly bound state.

Denoting the concentrations of the freely diffusing population, the weakly bound population and the strongly bound population by *u*, *w*, and *v*, respectively, we express any model in Fig 1 as a system of reaction—diffusion equations. Therefore, the reaction—diffusion equations for the model that includes all the possible interactions among these populations (model 1 in Fig 1) are given by

$$\begin{array}{cc} u_{t}= & D\Delta u-\gamma_{b}u+\gamma_{u}w-\kappa_{b}u+\kappa_{u}v\\ w_{t}= & \;-\eta_{b}w+\eta_{u}v+\gamma_{b}u-\gamma_{u}w\\ v_{t}= & \;+\kappa_{b}u-\kappa_{u}v+\eta_{b}w-\eta_{u}v \end{array}$$ (1)

where *γ*_*b*_ (*γ*_*u*_) denotes the transition rate from (to) a freely diffusing state to (from) a weakly bound state, *η*_*b*_ (*η*_*u*_) denotes the transition rate from (to) a weakly bound state to (from) a strongly bound state, and *κ*_*b*_ (*κ*_*u*_) denotes the transition rate from (to) a freely diffusing state to (from) a strongly bound state. The other sub-models (’nested’ models to the three-population model) can be expressed similarly as sub-cases of this system of reaction—diffusion equations by simply assuming one or more exchange rates to be equal to zero.

We use all the resulting mathematical models to fit experimental FRAP data coming from six different identified and described variants of histone H1, namely H1.0, H1.1, H1.2, H1.3, H1.4 and H1.5 [6]. The differences in the amino acid sequence of the N- and C-terminal domains of the variants can determine functional differences in chromatin binding. Thus, the reason for considering these different variants of histone H1 is to find out if the difference in their amino acid sequence, functionality, affinity for chromatin, and promotion of chromatin condensation [20, 21] has an effect on the assembly pathway.

Before carrying out the fitting, the models are solved explicitly in order to provide an analytical expression for the solution and a theoretical FRAP recovery curve to fit the data. The importance of having an analytical expression for the solution that facilitates the computational task of fitting a model to FRAP data has been strongly stressed even for a simple system of two reaction—diffusion equations [11–13, 22, 23]. Thus, it becomes an equally relevant, and slightly more complicated task that of finding an analytical expression for the solutions to our systems involving three reaction-diffusion equations.

We will use nonlinear least squares to fit the models to the data and assess and compare the models efficiency to describe the experimental data. This model comparison analysis, together with some model inference, will allow us to select the model (or models) that better describes the assembly pathway of each of these variants of histone H1 to chromatin.

## Results

### A theoretical FRAP curve for the histone H1 chromatin assembly pathway models

In order to obtain a theoretical FRAP curve to fit the data based on all the pathways described in Fig 1, we solved the system of reaction—diffusion Eq (1) explicitly. For this purpose, we assumed a narrow band of width 2*h*, centered at *c* on an approximated nucleus of length *L*. After going through all the mathematical analysis and calculations to find an analytical expression for the solution of the reaction—diffusion system given by Eq (1), we obtained the following theoretical recovery curve to fit FRAP data (see Models section for details):

$$\begin{array}{c} F\left(t\right)=1-\frac{L^{2}}{\left(L-2h\right)h\Pi_{total}}\sum_{n=1}^{\infty }[\left(\Pi_{total}+\Sigma_{u}D{\left(\frac{n\pi }{L}\right)}^{2}\right)a_{n}\left(t\right)\;\\ +\left(\Sigma_{total}+D{\left(\frac{n\pi }{L}\right)}^{2}\right)a_{n}^{\prime }\left(t\right)+a_{n}^{\prime \prime }\left(t\right)]S_{n}^{2} \end{array}$$ (2)

where

$$\begin{array}{c} \Sigma_{total}=\kappa_{b}+\gamma_{b}+\Sigma_{u},\;\Sigma_{u}=\kappa_{u}+\gamma_{u}+\eta_{b}+\eta_{u},\;\Pi_{total}=\Pi_{u}+\Pi_{w}+\Pi_{v} \end{array}$$

$$\begin{array}{c} \Pi_{u}=\kappa_{u}\eta_{b}+\kappa_{u}\gamma_{u}+\eta_{u}\gamma_{u},\;\Pi_{w}=\gamma_{b}\kappa_{u}+\gamma_{b}\eta_{u}+\kappa_{b}\eta_{u},\;\Pi_{v}=\eta_{b}\gamma_{b}+\eta_{b}\kappa_{b}+\gamma_{u}\kappa_{b} \end{array}$$

$$\begin{array}{c} S_{n}=\frac{1}{n\pi }[\sin(\frac{n\pi (c-h)}{L})-\sin(\frac{n\pi (c+h)}{L})]\; \end{array}$$

and the roots *z*_1*n*_, *z*_2*n*_ and *z*_3*n*_ of the polynomials

$$\begin{array}{c} r_{n}\left(s\right)=s^{3}+\left(\Sigma_{total}+D{\left(\frac{n\pi }{L}\right)}^{2}\right)s^{2}+\left(\Pi_{total}+\Sigma_{u}D{\left(\frac{n\pi }{L}\right)}^{2}\right)s+\Pi_{u}D{\left(\frac{n\pi }{L}\right)}^{2} \end{array}$$

determine the functions

$$\begin{array}{c} a_{n}\left(t\right)=\{\begin{array}{cc} A_{1n}e^{z_{1n}t}+A_{2n}e^{z_{2n}t}+A_{3n}e^{z_{3n}t} & \text{if}\;z_{1n},z_{2n},z_{3n}\in R\;\\ 2|A_{1n}|e^{\sigma_{1n}t}\left(\cos\left(\omega_{n}t+\arg\left(A_{1n}\right)\right)\right)+A_{3n}e^{z_{3n}t} & \text{if}\;z_{1n}=\bar{z_{2n}}\in C\;\setminus \;\;R \end{array} \end{array}$$

where

$$\begin{array}{c} A_{in}=\frac{{\left(z_{in}\right)}^{2}+\Sigma_{total}z_{in}+\Pi_{total}}{\prod_{j\neq i}\left(z_{in}-z_{jn}\right)},\;\text{for}\;i=1,2,3\;. \end{array}$$

Note that all the theoretical recovery curves corresponding to all the pathways in Fig 1 can be obtained from Eq (2) by simply assuming one or more of the exchange rates to be equal to zero.

### Fitting results

**Fig 2:**
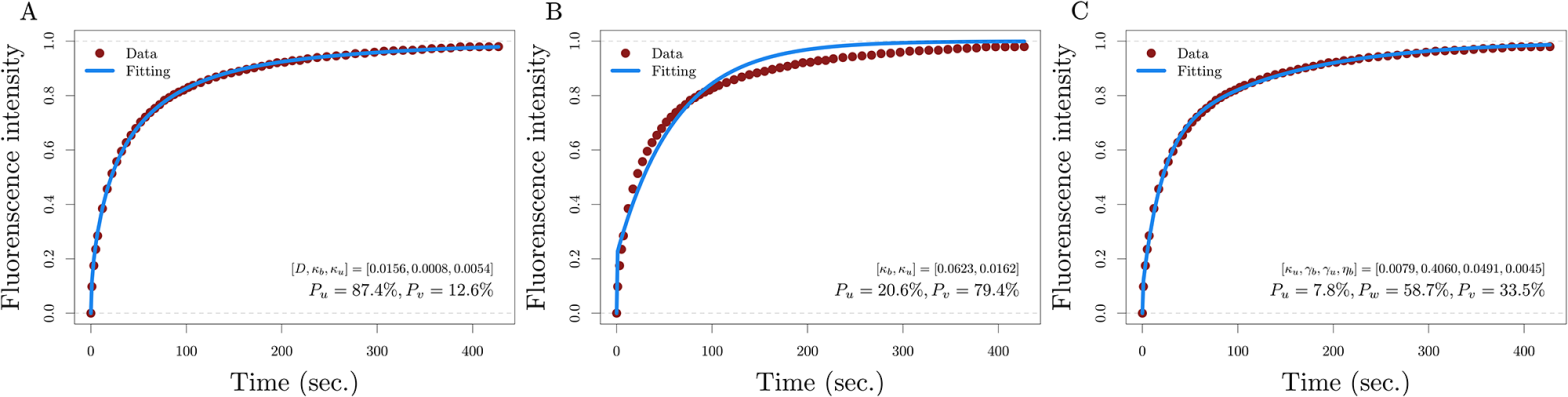
Sample of fitting results. Fitting of H1.2 data with A) the two population model 0 with a diffusion coefficient *D* as a parameter, B) the two population model 0 with fixed diffusion coefficient *D* = 25 μm^2^/s, and C) the three population model 9 (a similar profile for the fitting is obtained with models 1 through 8).

We fitted the corresponding theoretical recovery curves of all models in Fig 1 to experimental FRAP data from H1.0, H1.1, H1.2, H1.3, H1.4, and H1.5 variants of histone H1. The experimental FRAP data used for the fitting was previously reported in Raghuram et al. [10]. This data was obtained by photobleaching a narrow band of width 1.5 m on cell nuclei of mouse embryonic fibroblasts and recording the fluorescence recovery every five seconds for the first ten data points, every ten seconds for the following ten data points, every fifteen seconds for the next twenty data points and then every twenty seconds until the final time. Details of the experimental procedures can be found in [10]. To illustrate the results of the fitting and the importance of considering a three-population model for an accurate and biologically meaningful fitting, we show in Fig 2 how the fitting of the commonly used two-population model compares to the fitting of one of the three-population models that accounts explicitly for both weakly and strongly bound populations to the chromatin.

Notice that the fitting of the two-population model (where three parameters, including the diffusion coefficient, are estimated) to the histone H1.2 data results in estimates of *D* = 0.0156 μm^2^/s, *P*_*u*_ ≈ 87% and *P*_*v*_ ≈ 13% for the diffusion coefficient, the proportion of histone H1 population moving freely, and the proportion of chromatin-bound histone H1, respectively. Even though the fitting is very accurate, these estimates show a much lower proportion of bound population and diffusion coefficient than biologically expected.

On the one hand, one could simply interpret the estimated proportion of diffusing population in the two-population model as accounting implicitly for both a weakly bound population and a freely diffusing population. We know already that this implicit consideration of weakly bound population used in previous research does offer a satisfactory interpretation, but does not offer, however, all the dynamical information of the weakly bound population [11, 12].

On the other hand, one might think that fixing the diffusion coefficient -to a value based on the molecular weight and density of the medium- in the two population model can provide a solution. By doing this, we see in Fig 2B that despite obtaining a much better estimate of the proportion of the histone H1 population bound to the chromatin (*P*_*b*_ ≈ 79%), the fitting looses accuracy significantly. It is worthwhile to mention that these results for the two population model also hold for the other variants of histone H1.

Therefore, in order to enrich the understanding of the experimental data and assembly pathway of histone H1, we include explicitly a weakly bound population in the analysis. The importance of this explicit consideration will become apparent using perturbation analysis after the model comparison analysis and selection (see the section of the Results on Perturbation analysis).

When fitting all the three-population models in Fig 1 to the experimental data, we fixed the value of the diffusion coefficient (*D* = 25 μm^2^/s) and obtained very accurate fittings with considerably small RSS (Residual Sum of Squares) values. This theoretical approximation for the diffusion coefficient, which can be obtained using the molecular weight of the fusion protein GFP-histone H1, the nuclear viscosity of its transport medium, the Einstein-Smoluchowski relation together with the Strokes’ law, has been reported to have values between 20 μm^2^/s and 40 μm^2^/s [12, 13, 22, 24, 25].

Note from Fig 1 that model 1 involves six transition rates whereas models 2 through 5 involve five and models 6 through 9 involve four transition rate parameters. We apply non-linear least squares with constrains to fit the models (i.e, their corresponding theoretical curves, obtained with Eq (2)) to the data. Specifically, we find the best parameter estimates within a parameter region of values not greater than two orders of magnitude (transition times greater than ten milliseconds) by running the least squares method numerically from 1000 random initial parameter values exponentially distributed with rate parameter *λ* = 0.8, and take the point(s) of convergence with the lowest RSS. Results on the best estimates when the rapid transition times are less than ten milliseconds are discussed later in the Perturbation analysis section.

Interestingly, we found that having more than four transition rates (models 1 through 5 in Fig 1) leads to overfitting; in particular, we obtain that extra parameters are numerically zero (see multiplefitH1?.R files in S1 File). This rules out models 1 through 5 as assembly pathway models describing the experimental data and leaves us with models 6 through 9 (i.e., models with only four transition rate parameters) as the candidate models for describing the assembly pathway of histone H1.

For these models, we found the best estimates to be unique within the parameter region considered. This is illustrated in Table 1 for histone H1.2 (the results for the other histone H1 variants can be found in the fittingResults.R file of the S1 File). Also, the corrected Akaike’s Information Criterion (AIC) [26] for these models was found to be the same (−631.3231 for H1.0 type, −566.8368 for H1.1 type, −591.9987 for H1.2 data, −556.7751 for H1.3, −579.9953 for H1.4 data, and −585.0421 for H1.5 data). Thus, models 6 through 9 in Fig 1 are equally feasible for describing the assembly pathway for histone H1 with chromatin. Thus, we were unable to select a best model among them using statistical methods. The next step in our attempt to refine the selection of a feasible assembly pathway for histone H1 among models 6 through 9, is to carry out a model inference analysis by looking closely at the meaning of the biological features of each model that resulted from the estimated parameters.

**Table 1:** Numerical fitting results of models 6 through 9 to histone H1.2 data.

Model	RSS	Conv.	Est.	*κ*_*b*_	*κ*_*u*_	*γ*_*b*_	*γ*_*u*_	*η*_*b*_	*η*_*u*_
6	2.232 ⋅ 10^−3^	20	$\hat{\theta }$	–	–	0.40596	0.04912	0.00380	0.00867
			$\hat{\epsilon }$	–	–	3.62 ⋅ 10^−2^	1.58 ⋅ 10^−3^	2.22 ⋅ 10^−4^	2.19 ⋅ 10^−4^
7	2.232 ⋅ 10^−3^	38	$\hat{\theta }$	0.04024	0.00794	0.36572	0.05365	–	–
			$\hat{\epsilon }$	3.61 ⋅ 10^−3^	1.76 ⋅ 10^−4^	3.29 ⋅ 10^−2^	1.78 ⋅ 10^−3^	–	–
8	2.232 ⋅ 10^−3^	52	$\hat{\theta }$	0.03429	–	0.37168	0.05365	–	0.00794
			$\hat{\epsilon }$	3.15 ⋅ 10^−3^	–	3.33 ⋅ 10^−2^	1.78 ⋅ 10^−3^	–	1.76 ⋅ 10^−4^
9	2.232 ⋅ 10^−3^	26	$\hat{\theta }$	–	0.00794	0.40596	0.04912	0.00453	–
			$\hat{\epsilon }$	–	1.76 ⋅ 10^−4^	3.62 ⋅ 10^−2^	1.58 ⋅ 10^−3^	2.62 ⋅ 10^−4^	–

Column ‘Conv.’ indicates the number of times the estimated parameter $\hat{\theta }$ was obtained out of the 1000 random initial values exponentially distributed with rate parameter *λ* = 0.8. Column ‘Est.’ through column ‘*η*_*u*_’ indicate the estimated values for the parameters with their corresponding standard errors $\hat{\epsilon }$. Parameters have units s^−1^. Note that these estimates can be used to verify the values in Fig 2.

### Model inference

Using the estimated transition rates in all models for all the histone variants, we were able to calculate the proportions of the population that are free to diffuse, weakly bound and strongly bound to the chromatin, and the corresponding average transition times among these different kinetic states of histone H1 (Table 2). This information allows us to identify both similar and different biological features among the most favorable models.

**Table 2:** Biological information obtained from fitting models 6 through 9 to histone H1.1-H1.5 data.

Model	*P*_*u*_	*P*_*w*_	*P*_*v*_	*τ*_*u*→*v*_	*τ*_*v*→*u*_	*τ*_*u*→*w*_	*τ*_*w*→*u*_	*τ*_*w*→*v*_	*τ*_*v*→*w*_
**Histone H1.0 data**									
6	11.8	48.3	39.9	–	–	14.86	60.64	439.74	362.91
7	11.8	35.0	53.2	93.85	421.38	17.66	52.23	–	–
8	11.8	41.6	46.6	107.13	–	17.26	52.23	–	421.38
9	11.8	41.6	46.6	–	421.38	14.86	60.64	376.46	–
**Histone H1.1 data**									
6	11.6	61.2	27.1	–	–	7.88	41.47	627.71	278.28
7	11.6	52.2	36.2	96.36	299.62	8.58	38.52	–	–
8	11.6	56.9	31.5	110.58	–	8.48	38.52	–	299.62
9	11.6	56.9	31.5	–	299.62	7.88	41.47	540.82	–
**Histone H1.2 data**									
6	7.8	64.1	28.1	–	–	2.46	20.36	263.26	115.28
7	7.8	52.9	39.3	24.85	125.92	2.73	18.64	–	–
8	7.8	58.7	33.5	29.17	–	2.69	18.64	–	125.92
9	7.8	58.7	33.5	–	125.92	2.46	20.36	220.70	–
**Histone H1.3 data**									
6	9.8	62.6	27.7	–	–	5.40	34.58	553.45	244.88
7	9.8	53.9	36.3	70.49	262.50	5.84	32.26	–	–
8	9.8	58.4	31.9	80.37	–	5.78	32.26	–	262.50
9	9.8	58.4	31.9	–	262.50	5.40	34.58	480.54	–
**Histone H1.4 data**									
6	9.9	59.1	31.0	–	–	11.10	66.54	828.34	434.15
7	9.9	48.8	41.3	113.27	474.71	12.31	60.85	–	–
8	9.9	54.1	36.0	129.93	–	12.14	60.85	–	474.71
9	9.9	54.1	36.0	–	474.71	11.10	66.54	712.24	–
**Histone H1.5 data**									
6	8.9	59.9	31.2	–	–	10.01	67.54	860.83	448.14
7	8.9	49.7	41.4	104.80	488.94	11.07	61.91	–	–
8	8.9	54.9	36.2	119.99	–	10.92	61.90	–	488.94
9	8.9	54.9	36.2	–	488.94	10.01	67.54	741.92	–

*P*_*u*_, *P*_*w*_ and *P*_*v*_ represent the proportions of histone H1 in a free to diffuse, weakly bound, and strongly bound states, respectively (see Eq (11)). *τ*_*u*→*w*_ and *τ*_*u*→*v*_ denote the average wandering times before binding in a weak or strong fashion, respectively; *τ*_*w*→*v*_ and *τ*_*v*→*w*_ denote the average transition times between weakly and strongly bound states; and *τ*_*w*→*u*_ and *τ*_*v*→*u*_ denote the average residence times in weakly and strongly bound states, respectively. These average times are found by inverting the value of the corresponding transition rates; for example, *τ*_*u*→*v*_ = 1/*κ*_*b*_.

In particular, notice that the freely diffusing proportion of the population is approximately 9% for all the histone variants regardless of the model used. Also, note that, except for H1.0, the proportion of the population that is weakly bound is always greater than the strongly bound, but with different ratios. Specifically, if the assembly pathway is described by model 6 then the weakly bound population is approximately twice as big as the strongly bound, whereas it would be only 1.2 or 1.5 times greater if the pathway were described with model 7 or models 8 and 9, respectively.

For each of the histone H1 variants, the average transient times describing the rapid interactions (*τ*_*u*→*w*_ and *τ*_*w*→*u*_) and the average residence time in the strongly bound state (*τ*_*v*→*w*_ and *τ*_*v*→*u*_) are similar in all models. However, among the variants, both the rapid interaction times and the average residence time in the strongly bound state are smaller for H1.2. This might be due to the shorter C-terminal domain in the H1.2 variant.

Interestingly, the proportions of strongly bound population in H1.1, H1.2 and H1.3 subtypes is considerably smaller than the same proportion in H1.0, H1.4 or H1.5 subtypes. This correlates well with the classification of H1.1, H1.2, H1.3 as weak-intermediate condensers and H1.0, H1.4 and H1.5 as strong condensers [21].

Finally, notice that in models 6 and 9, the transition to the strongly bound state is slower than dissociation from that state, whereas in models 7 and 8 assembly to the strongly bound state is faster than dissociating from it. Although it is not clear to us how this difference could offer more insight for the purpose of refining our model selection, we don’t rule out the possibility that it may offer a clue for fully unfolding the assembly dynamics of histone H1 in future research. Notice as well that models 6 and 9 seem to be consistent with the findings of the cooperative role that the low affinity H1 binding has in the transition to a high affinity H1 binding [10, 15, 27]. However, this is not a strong argument for refining our model selection.

Even though the model inference offers an interesting insight into the subtypes classification of histone H1, the analysis does not allow us to further refine the model selection.

### Perturbation analysis

A natural question that arises when considering a three-population model to describe the spatio-temporal dynamics of histone H1 is whether or not this model can be reduced to a two-population model in which the weak interaction is taken implicitly (within an effectively diffusing population that comprises both the freely diffusing and the weakly bound population) and the strong interaction is taken explicitly. We prove, using perturbation analysis, that even though this simplification is possible under certain conditions, the explicit consideration of both weak and strong interactions enriches the interpretation of histone H1 FRAP experimental data.

Having reduced our selection to models 6 through 9 in Fig 1, we use model 9 to illustrate the results (the same analysis is carried out for models 6, 7 and 8 in the S3 File of the Supporting Information). The system of reaction-diffusion equations describing such a model is given by

$$\begin{array}{cc} \frac{\partial }{\partial t}u\left(x,t\right) & =D\frac{\partial^{2}}{\partial x^{2}}u\left(x,t\right)-\gamma_{b}u\left(x,t\right)+\gamma_{u}w\left(x,t\right)+\kappa_{u}v\left(x,t\right),\\ \frac{\partial }{\partial t}w\left(x,t\right) & =\;+\gamma_{b}u\left(x,t\right)-\gamma_{u}w\left(x,t\right)-\eta_{b}w\left(x,t\right),\\ \frac{\partial }{\partial t}v\left(x,t\right) & =\;-\kappa_{u}v\left(x,t\right)+\eta_{b}w\left(x,t\right). \end{array}$$ (3)

Assuming that $\gamma_{b}=\frac{\lambda_{b}}{\varepsilon }$, and $\gamma_{u}=\frac{\lambda_{u}}{\varepsilon }$, where *ε* << 1 (i.e., the turnover of weakly bound biomolecules into a freely diffusing state is sufficiently fast), and carrying out a perturbation analysis, we prove (see Models section for details) that the reaction-diffusion system (3) can be approximated with its following leading-order reaction-diffusion system of two equations

$$\begin{array}{cc} \frac{\partial }{\partial t}c_{0}\left(x,t\right) & =\frac{D}{1+\gamma }\frac{\partial^{2}}{\partial x^{2}}c_{0}\left(x,t\right)+\kappa_{u}v_{0}\left(x,t\right)-\eta_{b}\frac{\gamma }{1+\gamma }c_{0}\left(x,t\right),\\ \frac{\partial }{\partial t}v_{0}\left(x,t\right) & =\;-\kappa_{u}v_{0}\left(x,t\right)+\eta_{b}\frac{\gamma }{1+\gamma }c_{0}\left(x,t\right), \end{array}$$ (4)

where *c*_0_(*x*, *t*) = *u*_0_(*x*, *t*) + *w*_0_(*x*, *t*); *u*_0_(*x*, *t*), *w*_0_(*x*, *t*) and *v*_0_(*x*, *t*) denote the leading-order approximations for *u*(*x*, *t*), *w*(*x*, *t*) and *v*(*x*, *t*), respectively, and $\gamma =\frac{\lambda_{b}}{\lambda_{u}}=\frac{\gamma_{b}}{\gamma_{u}}$.

Thus, we conclude that if the turnover of weakly bound biomolecules to/from a freely diffusing state is sufficiently fast (in other words, if the assumption of high turnover rates *γ*_*b*_ and *γ*_*u*_ is met) the reaction-diffusion system of three Eq (3) can be approximated with the reaction-diffusion system of two Eq (4).

We now show, using this result, that to fully describe the dynamics of histone H1 within a certain region of the parameter space, it is necessary to account explicitly for for three populations (freely diffusing, weakly chromatin-bound, and strongly chromatin-bound) as described, for example, by the system of reaction-diffusion Eq (3), and that this dynamics cannot be approximated with the reaction-diffusion system of Eq (4). In other words, the turnover of weakly bound biomolecules into a freely diffusing state is not sufficiently fast to make such an approximation.

**Fig 3:**
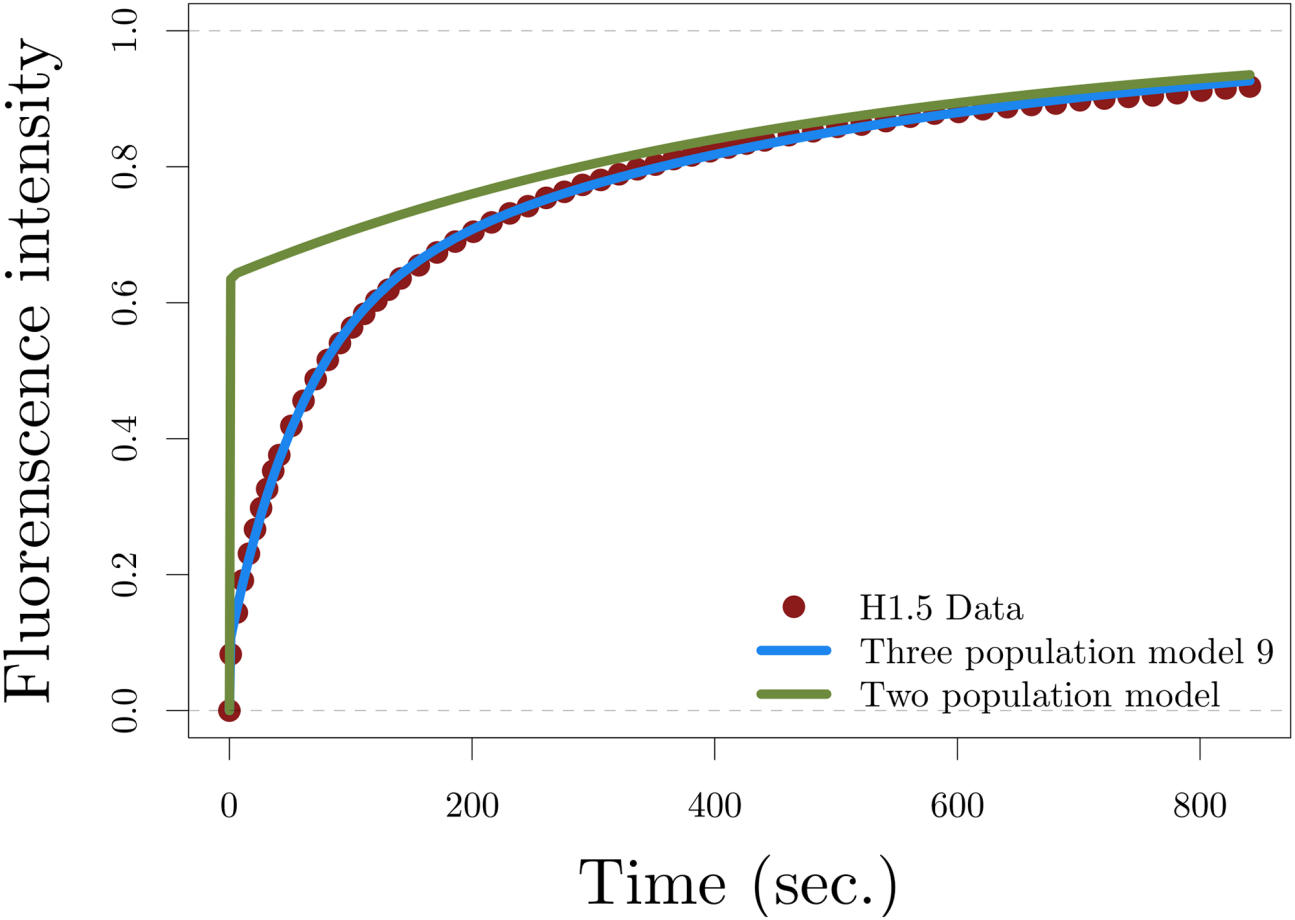
Validating the three population model. Fitting the three-population model to histone H1.5 data (blue) and sketch of the solution of the approximated two population model given by Eq (4) with the parameter values in Eq (6) (green).

In particular, if we use the solution of the reaction-diffusion system of Eq (3) to fit histone H1.5 data assuming a diffusion coefficient *D* = 25 μm^2^/s, we obtain parameter values of

$$\begin{array}{c} \kappa_{u}=0.002045/\sec,\;\gamma_{b}=0.099881/\sec,\;\gamma_{u}=0.014805/\sec,\;\eta_{b}=0.001347/\sec, \end{array}$$ (5)

and an accurate fitting as shown in Fig 3.

If the turnover of weakly bound biomolecules to a freely diffusing state described by the parameter $\gamma =\frac{\gamma_{b}}{\gamma_{u}}$ was sufficiently fast then the solution of the reaction-diffusion system of two Eq (4) with the parameter values given by

$$\begin{array}{c} \frac{D}{1+\gamma }=3.227462443{\mu \text{m}}^{2}/\text{s},\;\eta_{b}\frac{\gamma }{1+\gamma }=0.001173848/\sec,\;\kappa_{u}=0.002045244/\sec, \end{array}$$ (6)

would also offer an accurate fitting to the data. However, as illustrated in Fig 3, this is not the case. Specifically, the leading-order approximation of the reaction—diffusion Eq (3), given by the system of two Eq (4), fails at describing accurately the effective diffusing phase of the recovery curve. Thus, the rapid interaction, described by the parameter *γ*, is not sufficiently fast for approximating the dynamics of histone H1 with its leading-order approximation. This fact offers a validation of the three population model as an essential tool for interpreting experimental histone H1 data. This same result is obtained for all variants of histone H1.

It is important to note that if one allows for values greater than two orders of magnitude for the parameter estimates, the leading-order approximation of model 9 can describe the effective diffusing phase of the recovery curve. In particular, if we fit directly the leading-order approximation (4) to the same histone H1.5 data, we obtain parameter values of

$$\begin{array}{c} \frac{D}{1+\gamma }=0.004768222{\mu \text{m}}^{2}/\text{s},\;\eta_{b}\frac{\gamma }{1+\gamma }=0.0002123/\sec,\;\kappa_{u}=0.0011232/\sec. \end{array}$$ (7)

**Fig 4:**
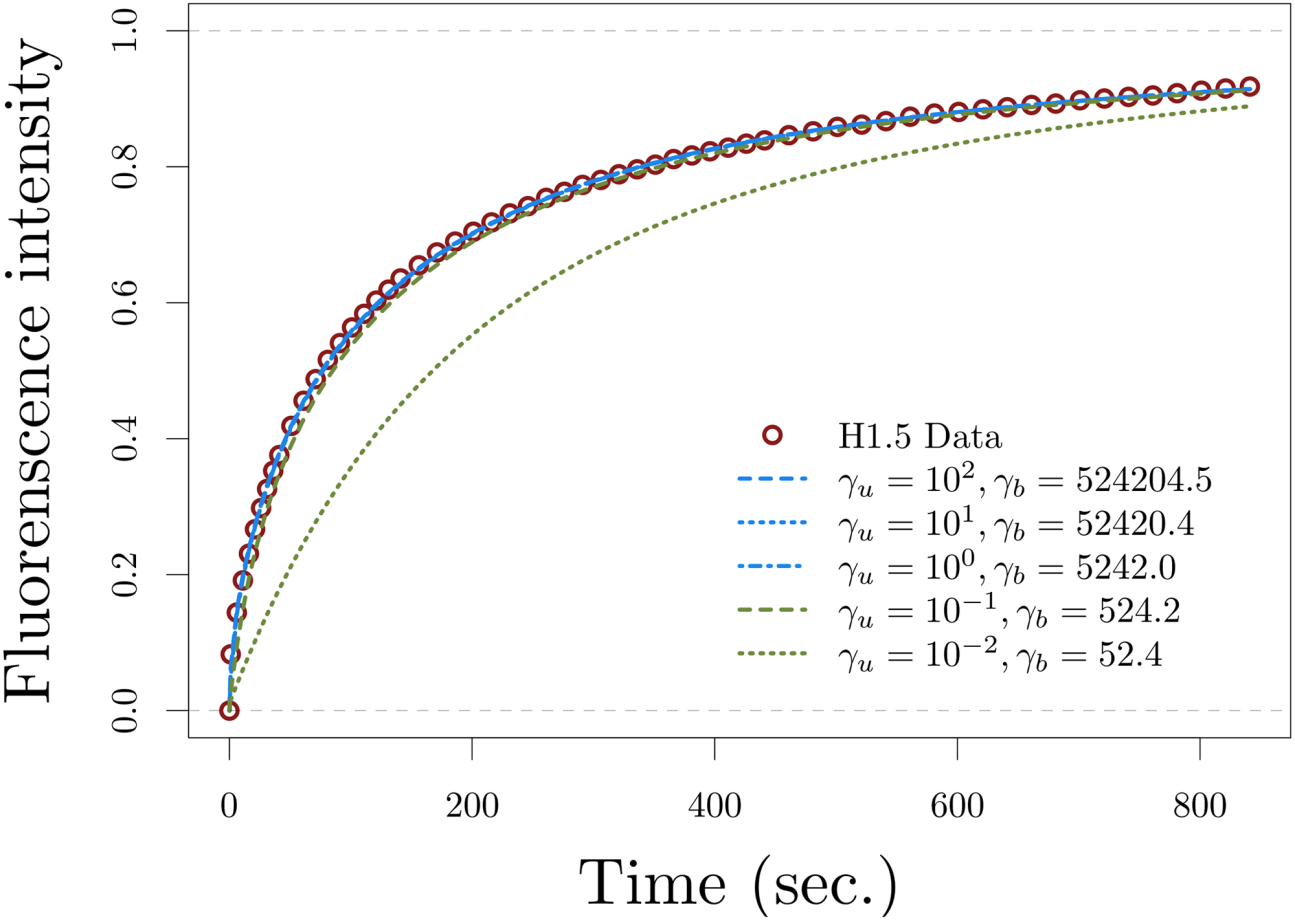
Recovery curves of the three population model 9 approximating H1.5 data for several parameter estimates with values greater than two orders of magnitude. The parameters values used for *γ*, *η*_*b*_, and *κ*_*u*_ (8) are the ones obtained from fitting the leading-order approximation (4) to the data, the values for *γ*_*u*_ and *γ*_*b*_ = *γγ*_*u*_ vary across different orders of magnitude, and *D* = 25 μm^2^/s. Note that when the order of magnitude of *γ*_*b*_ is two (green dotted) or less, model 9 and its leading-order approximation are no longer equivalent.

Assuming a diffusion coefficient *D* = 25 μm^2^/s, we can estimate the following parameters for model 9

$$\begin{array}{c} \kappa_{u}=0.0011232/\sec,\;\gamma =\frac{D}{0.004768222}-1=5242.045,\;\eta_{b}=0.0002123/\sec; \end{array}$$ (8)

however, *γ*_*b*_ and *γ*_*u*_ cannot be uniquely determined. This is illustrated in Fig 4, where we used these parameter estimates to sketch the explicit solution of the full model 9 for different values of *γ*_*u*_ and *γ*_*b*_, keeping their ratio *γ* fixed.

**Fig 5:**
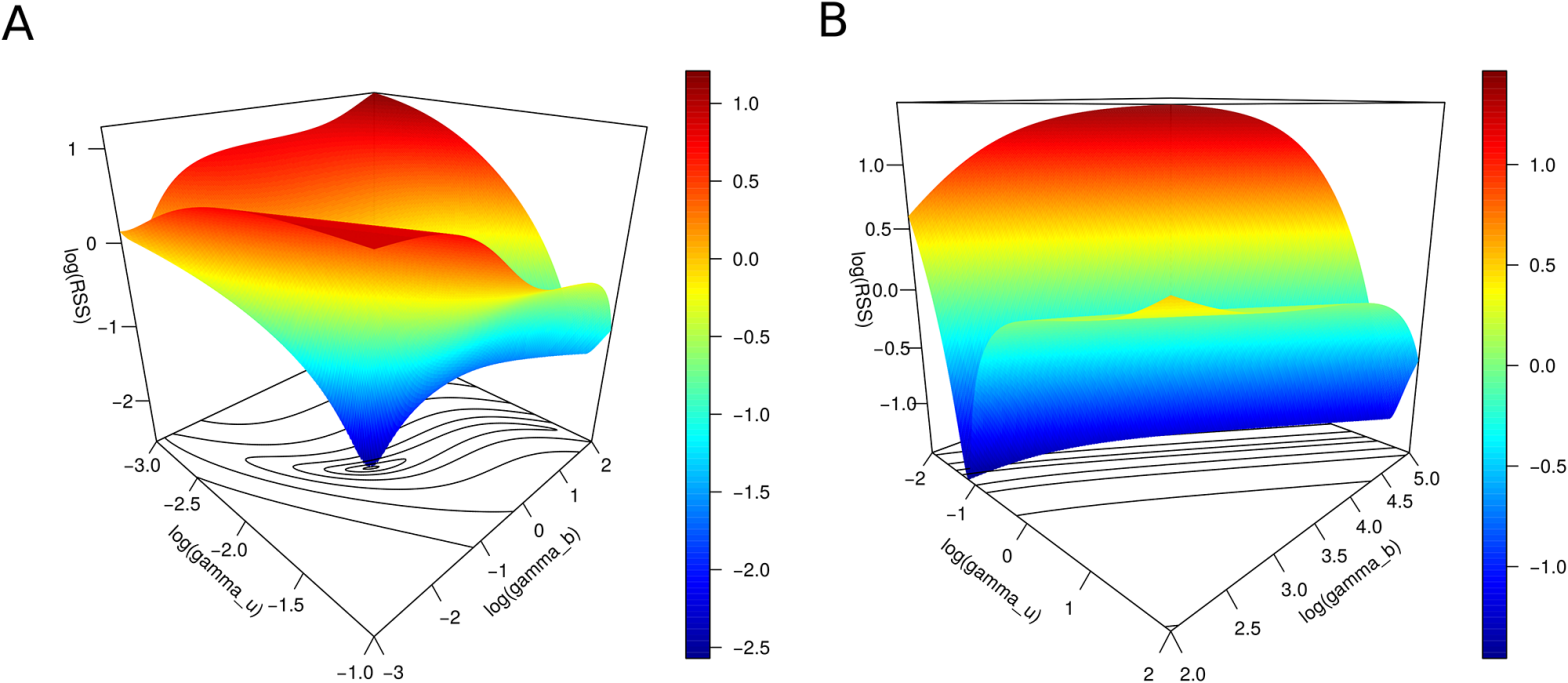
Surface plots of the RSS versus *γ*_*b*_ and *γ*_*u*_ for H1.5 data and model 9. Since the parametric space is four dimensional, these plots are projections onto the *γ*_*b*_ − *γ*_*u*_ subspace. A) RSS when the parameter region is restricted to values not greater than two orders of magnitude. B) RSS when the parameter region accepts values greater than two orders of magnitude.

Therefore, if the three population model 9 can be approximated by its leading-order approximation, the rapid interaction is characterized only by the ratio *γ* as the particular values for *γ*_*b*_ and *γ*_*u*_ could not be uniquely determined. This difference from the case with parameter values not greater than two orders of magnitude, where both *γ*_*b*_ and *γ*_*u*_ can be uniquely determined, is illustrated in Fig 5, the surface plot of the RSS with respect *γ*_*b*_ and *γ*_*u*_ obtained from fitting model 9 to the histone H1.5 data. Fig 5A shows that the RSS has a unique minimum when the three population model is fitted to the data and the parameter region is restricted to values not greater than two orders of magnitude (this corresponds to the accurate fitting in Fig 3), and Fig 5B shows a continuum of minima for the RSS when the three population model is fitted to the data and the parameter region allows for values greater than two orders of magnitude (this corresponds to the different accurate fittings in Fig 4).

Table 3 contains the biological information from the parameter estimates obtained when fitting the leading-order approximation of models 6 through 9 to all the variants of histone H1 data. Note that some information on the kinetics of the assembly pathway cannot be obtained when using the leading-order approximation.

**Table 3:** Biological information obtained from fitting the leading-order approximation of models 6 through 9 to histone H1.1-H1.5 data.

Model	*P*_*u*_	*P*_*w*_	*P*_*v*_	*τ*_*u*→*v*_	*τ*_*v*→*u*_	*τ*_*u*→*w*_	*τ*_*w*→*u*_	*τ*_*w*→*v*_	*τ*_*v*→*w*_
**Histone H1.0 data**									
6	0.021	63.361	36.618	–	–	–	–	693.49	400.78
7	0.021	63.361	36.618	0.23	400.78	–	–	–	–
8	–	–	–	0.23	–	–	–	–	400.78
9	–	–	–	–	400.78	–	–	693.49	–
**Histone H1.1 data**									
6	0.032	80.362	19.606	–	–	–	–	1468.59	358.29
7	0.032	80.362	19.606	0.58	358.29	–	–	–	–
8	–	–	–	0.58	–	–	–	–	358.29
9	–	–	–	–	358.29	–	–	1468.59	–
**Histone H1.2 data**									
6	0.055	87.331	12.614	–	–	–	–	1279.41	184.80
7	0.055	87.331	12.614	0.80	184.80	–	–	–	–
8	–	–	–	0.80	–	–	–	–	184.80
9	–	–	–	–	184.80	–	–	1279.41	–
**Histone H1.3 data**									
6	0.035	81.509	18.456	–	–	–	–	1454.10	329.25
7	0.035	81.509	18.456	0.62	329.25	–	–	–	–
8	–	–	–	0.62	–	–	–	–	329.25
9	–	–	–	–	329.25	–	–	1454.10	–
**Histone H1.4 data**									
6	0.017	79.336	20.647	–	–	–	–	2429.18	632.18
7	0.017	79.336	20.647	0.52	632.18	–	–	–	–
8	–	–	–	0.52	–	–	–	–	632.18
9	–	–	–	–	632.18	–	–	2429.18	–
**Histone H1.5 data**									
6	0.016	84.088	15.896	–	–	–	–	4709.88	890.33
7	0.016	84.088	15.896	0.90	890.33	–	–	–	–
8	–	–	–	0.90	–	–	–	–	890.33
9	–	–	–	–	890.33	–	–	4709.88	–

One can uniquely determine *γ* = *γ*_*b*_/*γ*_*u*_ by fixing *D* = 25 μm^2^/s. However, the estimates for *γ*_*b*_ and *γ*_*u*_ are not unique; therefore, the parameter estimates shown in the table are computed when possible. For instance, the proportions of freely diffusing, weakly bound, and strongly bound populations for models 6 and 7 can be found from the ratio *γ*, but this is not the case for models 8 and 9. The description of the estimated parameters is found in Table 2.

### Application: Effect of core histone hyperacetylation on the association of histone H1 with chromatin

In this section, we illustrate an application by using the three-population model 9 to assess the effect of core histone hyperacetylation on the binding affinity of histone H1 with chromatin.

Hyperacetilation is a post-traslational modification known to alter histone H1 binding affinity with chromatin [7, 10, 28, 29]. In particular, Raghuram et al. established the the effect of TSA (Trichostatin-A) on the binding affinity of histone H1 with chromatin [10]. TSA is a histone deacetylase inhibitor that causes hyperacetylation and chromatin decondensation.

In this section, we use the experimental FRAP data reported previously in Raghuram et al. [10] to assess the effect of core histone hyperacetylation. Briefly, this data consists of three groups for all histone H1 types. The first set of FRAP data is a control group obtained after photobleaching a narrow band of width 1.5 m on cell nuclei of mouse embryonic fibroblasts; the second set of FRAP data is obtained after photobleaching a narrow band of width 1.5 m on cell nuclei of mouse embryonic fibroblasts treated with TSA for one hour; and the third set of FRAP data is obtained after photobleaching a narrow band of width 1.5 m on cell nuclei of mouse embryonic fibroblasts treated with TSA for 18 hours.

For the purpose of illustrating this application, the three population model 9 in Fig 1 was fitted to each group of FRAP data for all histone H1 variants assuming that transition times between states are greater than ten milliseconds. In other words, we estimated the parameters *γ*_*b*_, *γ*_*u*_, *η*_*b*_, *κ*_*u*_ describing all the transition rates involved in the model for all the control cells, the cells treated with TSA for 1 hour, and the cells treated with TSA for 18 hours, for all histone H1 variants. Using these estimates, we identified their significant changes from the control group to the TSA treatment groups (1 hour and 18 hours) for each of the histone H1 variants using the nonparametric K-S test (Kolmogorov-Smirnov test) at 5% significance level. The p-values are shown in Table 4. Even though it is not specified in the table, all the kinetic parameters that changed significantly did so by increasing their value after TSA treatment, or equivalently, all the transitions or interactions described by these parameters became significantly faster.

**Table 4:** P-values of the K-S test for the kinetic parameter changes after TSA treatment for all histone H1 variants.

	1 hr				18 hrs			
	*κ*_*u*_	*γ*_*b*_	*γ*_*u*_	*η*_*b*_	*κ*_*u*_	*γ*_*b*_	*γ*_*u*_	*η*_*b*_
H1.0	.502	.721	.093	.750	.**000**	.**005**	.**000**	.179
H1.1	.660	.**028**	.660	.375	.**007**	.**001**	.**000**	.503
H1.2	.210	.210	.699	.415	.983	.**032**	.**024**	.221
H1.3	.324	.517	.517	.928	.**001**	.**002**	.**000**	.053
H1.4	.948	.759	.635	.869	.**003**	.**002**	.**000**	.**030**
H1.5	.199	.730	.075	.069	.163	.332	.**004**	.281

Transition rates that changed significantly after TSA treatment (1 hr and 18 hours) on the basis of the K-S test at 5% significance level are marked in bold fond.

One can notice from Table 4 that in general there is no significant change in the chromatin binding affinity of histone H1 after 1 hour of TSA treatment and that all the transition rates, with the exception of *η*_*b*_, changed significantly after 18 hours of TSA treatment. In other words, the transition of histone H1 from a low affinity chromatin binding to a high affinity chromatin binding is not significantly affected by TSA treatment. This is an interesting result if one think of *η*_*n*_ as a kinetic parameter describing a cooperative behaviour to attain high affinity chromatin binding. Specifically, one could establish that hyperacetylation does not affect this cooperative aspect in the dynamics of histone H1. This result that is, surprisingly, opposite of the conclusion obtained by Raghuram et al. [10], could pass undetected if one did not interpret the data with a three population model.

Also, notice that TSA treatment after 18 hours does affect all other kinetic parameters that determine the chromatin binding affinity and the free and bound pools of histone H1. Note that in the case of H1.4 all kinetic parameters changed significantly after 18 hours of TSA treatment. Notice as well that in the case of H1.5 none of the kinetic parameters (except *γ*_*u*_) changed significantly after TSA treatment. This might be related to the long C-terminal domain of histone H1.5 and its strong condenser nature.

## Discussion

We have proposed nine models, expressed as systems of reaction—diffusion equations, to describe the chromatin assembly pathway for histone H1 based on both weakly and strongly bound interactions with the chromatin. By solving these models explicitly, we obtained a theoretical FRAP curve that can be used to fit histone H1 experimental FRAP data and perform a model comparison analysis. The results of the comparison analysis, favored four of the nine models (models 6–9 in Fig 1) as the ones that better describe the pathway of histone H1 assembly into chromatin.

As the assembly pathway described by these four models is different, we carried out an inferential analysis (Table 2), where the proportions of histone H1 diffusing, interacting weakly and strongly with the chromatin were estimated assuming that the transition times from each state to another are greater than ten milliseconds.

Even though the estimation of the proportions of histone H1 in each state is fairly consistent between the four models (approximately 55% for the weakly bound, 35% for the strongly bound and 10% for the freely diffusing), the average transition times from one state to another offered a quantitative difference between the dynamics of models 7 and 8 and models 6 and 9. In particular, for models 7 and 8 the average binding time is significantly lower than the unbinding time. Since there is no evidence that histone H1 binding requires more time than the unbinding, models 7 and 8 remained as feasible models describing the assembly pathway for histone H1. Interestingly, models 6 and 9 seem to be consistent with the finding that histone H1 progressively and cooperatively binds to the linker DNA [10, 15, 27], suggesting the existence of an intermediate weaker state before attaining a strongly bound state.

We notice in Table 2 that the average residence times and proportions in a strongly bound state for models 6 through 9 and all variants of histone H1 are consistent with the classification of histones H1.1, H1.2, and H1.3 as a weak-intermediate condenser, and histone H1.0, H1.4, and H1.5 as strong condensers [21]. For histone H1.2, the average residence time in a bound state (either weak or strong) is smaller than that of the other types of histone H1. This might be due to the short C-terminal domain of histone H1.2. Also, the results are consistent with H1.2 having the largest weakly bound population amongst the variants [30].

Thus, models 6 through 9 provide relevant biophysical quantitative information that is consistent with previous literature. Even though these models describe, on the basis of our analysis, equally feasible assembly pathways, one could be biased towards models 7 and 9. On the one hand, notice that the transition from the weakly bound state to the strongly one in model 6 is reversible; however, the “intrinsically unstructured” nature of histone H1 leads one to think that this transition is not likely to be reversible [6, 15]. A similar argument can be applied to the unlikely transition from a strongly bound state to a weakly one in model 8. On the the other hand, the observed “strong mutual repulsion” of linker DNA [31], which might be the result of a direct dissociation pathway—different from the cooperativity– affected mainly by ATP-dependent processes [15, 32], is consistent with models 7 and 9.

Whether there is a unique assembly pathway of histone H1 with the chromatin or not seems to remain an open question. It is possible, for instance, that histone H1 expresses different assembly and disassembly pathways within different regions of the cell nucleus (heterochromatin or euchromatin), which would require different models to describe and quantify histone H1 dynamics (i.e., a multi—model approach). The possibility of different pathways might be due to changes in histone variant or modification status, structural differences between forms of chromatin that alter binding sites, and/or post-translational modification of histone H1. However, having an overall description of the dynamics of histone H1 within the cell nucleus is essential for quantifying the biophysical parameters involved. We showed, using perturbation analysis, that this dynamic description is enriched with the explicit consideration of both high- and low-affinity associations of histone H1 with chromatin.

Our results provide, with models 6 through 9, four feasible assembly pathways describing the dynamics of histone H1. These pathways can be used to quantify explicitly not only the proportions of histone H1 weakly and strongly bound to chromatin, and free to diffuse, but also all the biophysical parameters involved in the transitions among these states.

Moreover, having a model describing explicitly the assembly pathway of histone H1 to chromatin provides a potential qualitative and quantitative tool to assess the effects of post-translational modifications on the association and dissociation dynamics of histone H1. To illustrate this, we applied the three population model 9 to assess the effect of core histone hyperacelylation on the binding affinity of histone H1. We showed that TSA treatment affects significantly all the kinetic binding interactions of histone H1 described by the model, except, surprisingly, the interaction represented by *η*_*b*_. As stated before, this is an interesting result if one think of *η*_*b*_ as a kinetic parameter describing a cooperative behaviour to attain high affinity binding. Specifically, the result can be used as a basis to establish that hyperacetylation does not affect significantly this cooperative aspect in the dynamics of histone H1. This conclusion is the opposite of the one obtained by Raghuram et al. in [10], where the authors claim that core histone hyperacetylation impacts cooperative behaviour of histone H1 to chromatin. We argue that this claim may be erroneous not only because a three population model was not considered but also because extrapolating that the cooperativity was altered based on the properties of individual histone H1 domains to hyperacetylation may not reflect the behaviour of the full length protein.

## Models

### Theoretical FRAP curve

We solved the reaction-diffusion system (1) to obtain the theoretical FRAP curve given by (2). Note that by considering a linear reaction in the model one assumes a homogeneous distribution of chromatin with permanent availability of binding sites. Also, due to the linear nature of the reaction in the system one can draw conclusions about the total population of proteins (photoactivated and non-photoactivated) by tracking only the photoactivated proteins. In particular, the linear reaction of the system together the uniqueness of its solution allows one to find the solution for the total population (photoactivated and non-photoactivated) by simply adding the solution of the system for the non-photoactivated proteins to the solution of the system for the photoactivated proteins after photobleaching. The photobleaching in the FRAP experiment, performed on a centered narrow band of a cell nucleus with an estimated length *L* allows us to reduce the spatial dimension of the problem to one dimension (details of such reduction can be found in [11]). Thus, the system of reaction—diffusion equations subject to no—flux boundary conditions describing the full three-population model (model 1 in Fig 1) is given by

$$\begin{array}{c} \begin{array}{cc} & \begin{array}{cc} \frac{\partial }{\partial t}u\left(x,t\right) & =D\frac{\partial^{2}}{\partial x^{2}}u\left(x,t\right)-\gamma_{b}u\left(x,t\right)+\gamma_{u}w\left(x,t\right)-\kappa_{b}u\left(x,t\right)+\kappa_{u}v\left(x,t\right)\;\\ \frac{\partial }{\partial t}w\left(x,t\right) & =\;-\eta_{b}w\left(x,t\right)+\eta_{u}v\left(x,t\right)+\gamma_{b}u\left(x,t\right)-\gamma_{u}w\left(x,t\right)\;\\ \frac{\partial }{\partial t}v\left(x,t\right) & =\;+\kappa_{b}u\left(x,t\right)-\kappa_{u}v\left(x,t\right)+\eta_{b}w\left(x,t\right)-\eta_{u}v\left(x,t\right)\; \end{array}\\ & x\in (0,L),\;t>0\\ & \frac{\partial }{\partial x}u\left(x,t\right)=\frac{\partial }{\partial x}w\left(x,t\right)=\frac{\partial }{\partial x}v\left(x,t\right)=0,\;x=0,L,\;t>0\\ & u(x,0)=f(x),\;w(x,0)=g(x),\;v(x,0)=h(x),\;x=0,L \end{array} \end{array}$$ (9)

where *D* is the diffusion coefficient, *κ*_*u*_, *κ*_*b*_, *γ*_*u*_, *γ*_*b*_, *η*_*u*_, *η*_*b*_ are transition rates among the possible histone H1 states. The initial condition is given by the functions

$$\begin{array}{c} \begin{array}{c} f\left(x\right)=\{\begin{array}{cc} 0 & \text{if}\;|x-c|\leq h\\ P_{u} & \text{if}\;|x-c|>h \end{array}\\ g\left(x\right)=\{\begin{array}{cc} 0 & \text{if}\;|x-c|\leq h\\ P_{w} & \text{if}\;|x-c|>h \end{array}\\ h\left(x\right)=\{\begin{array}{cc} 0 & \text{if}\;|x-c|\leq h\\ P_{v} & \text{if}\;|x-c|>h \end{array} \end{array} \end{array}$$ (10)

where *c* denotes the center of the photobleached narrow band of width 2*h* and the constants *P*_*u*_, *P*_*w*_ and *P*_*v*_ are the proportions of *u*(*t*, *x*), *w*(*t*, *x*) and *v*(*t*, *x*) at steady state, respectively. After some calculations it can be shown that

$$\begin{array}{c} P_{u}=\frac{\Pi_{u}}{\Pi_{total}},\;P_{w}=\frac{\Pi_{w}}{\Pi_{total}},\;P_{v}=\frac{\Pi_{v}}{\Pi_{total}} \end{array}$$ (11)

where

$$\begin{array}{c} \begin{array}{cc} \Pi_{u} & =\kappa_{u}\eta_{b}+\kappa_{u}\gamma_{u}+\eta_{u}\gamma_{u}\\ \Pi_{w} & =\gamma_{b}\kappa_{u}+\gamma_{b}\eta_{u}+\kappa_{b}\eta_{u}\\ \Pi_{v} & =\eta_{b}\gamma_{b}+\eta_{b}\kappa_{b}+\gamma_{u}\kappa_{b}\\ \Pi_{total} & =\Pi_{u}+\Pi_{w}+\Pi_{v} \end{array} \end{array}$$ (12)

Notice that the reaction—diffusion system of Eq (9) accounts for models 2 through 9 as these models can be obtained by setting the appropriate transition rates to zero. We obtain the theoretical FRAP curve by solving the system and integrating the sum of the three-population over the photobleached bandwidth. The following theorem provides the necessary conditions for obtaining a theoretical FRAP curve.

**Theorem 1** *Let the system* (9) *with initial conditions* (10) *and constants* (12). *If*

$$\begin{array}{c} \begin{array}{c} \kappa_{u}+\eta_{u}>0,\;\kappa_{b}+\eta_{b}>0,\;\kappa_{b}+\gamma_{b}>0,\\ \kappa_{u}+\gamma_{u}>0,\;\gamma_{b}+\eta_{u}>0,\;\gamma_{u}+\eta_{b}>0,\\ \kappa_{u}<\gamma_{u}, \end{array} \end{array}$$ (13)

*then the theoretical FRAP curve is given by the function* (2).

Notice that the first six conditions in (13) prevent the model from being senseless, i.e., from having a population class with no outgoing or no incoming rates. The last condition in (13) just ensures that the rapid interaction is described by the parameters *γ* in the system of equations.

To prove Theorem 1, we first apply Laplace transform to modify the problem of solving a system of partial differential equations into a problem of solving a system of ordinary differential equations. We reduce this system to a single second-order ordinary differential equation and express its solution as a Fourier series, where the coefficients are given by a third degree polynomial. Then, we apply the inverse Laplace transform to find the solution of the original system of partial differential equations describing the dynamics of histone H1, and finally, we integrate over the photobleached area to obtain the FRAP function *F*(*t*).

### Perturbation analysis. Leading order approximation

As mentioned in the Results, we use model 9 to illustrate its leading-order approximation system of two equations. An analogous perturbation analysis is carried out for models 6, 7 and 8 in the S3 File of the Supporting Information.

The system of reaction-diffusion equations describing model 9 is given by

$$\begin{array}{c} \begin{array}{cc} & \begin{array}{cc} \frac{\partial }{\partial t}u\left(x,t\right) & =D\;\frac{\partial^{2}}{\partial x^{2}}u\left(x,t\right)-\gamma_{b}u\left(x,t\right)+\gamma_{u}w\left(x,t\right)+\kappa_{u}v\left(x,t\right)\;,\\ \frac{\partial }{\partial t}w\left(x,t\right) & =\;+\gamma_{b}u\left(x,t\right)-\gamma_{u}w\left(x,t\right)-\eta_{b}w\left(x,t\right)\;,\\ \frac{\partial }{\partial t}v\left(x,t\right) & =\;-\kappa_{u}v\left(x,t\right)+\eta_{b}w\left(x,t\right)\;. \end{array} \end{array} \end{array}$$ (14)

Assuming that $\gamma_{b}=\frac{\lambda_{b}}{\varepsilon }$, and $\gamma_{u}=\frac{\lambda_{u}}{\varepsilon }$, where *ε* << 1 (i.e., the turnover of weakly bound biomolecules into a freely diffusing state is sufficiently fast), the reaction-diffusion system (14) can be rewritten as

$$\begin{array}{c} \begin{array}{cc} & \begin{array}{cc} \frac{\partial }{\partial t}\left(u\left(x,t\right)+w\left(x,t\right)\right) & =D\;\frac{\partial^{2}}{\partial x^{2}}u\left(x,t\right)+\kappa_{u}v\left(x,t\right)-\eta_{b}w\left(x,t\right)\;,\\ \varepsilon \frac{\partial }{\partial t}w\left(x,t\right) & =\;+\lambda_{b}u\left(x,t\right)-\lambda_{u}w\left(x,t\right)-\varepsilon \eta_{b}w\left(x,t\right)\;,\\ \frac{\partial }{\partial t}v\left(x,t\right) & =\;-\kappa_{u}v\left(x,t\right)+\eta_{b}w\left(x,t\right)\;. \end{array} \end{array} \end{array}$$ (15)

If we now consider a perturbation expansion for *u*(*x*, *t*), *w*(*x*, *t*) and *v*(*x*, *t*) of the form

$$\begin{array}{c} u\left(x,t\right)∼\sum_{n=0}^{\infty }\varepsilon^{n}u_{n}\left(x,t\right)\;,\;w\left(x,t\right)∼\sum_{n=0}^{\infty }\varepsilon^{n}w_{n}\left(x,t\right)\;,\;v\left(x,t\right)∼\sum_{n=0}^{\infty }\varepsilon^{n}v_{n}\left(x,t\right)\;, \end{array}$$ (16)

we obtain the following leading-order system for (15)

$$\begin{array}{c} \begin{array}{cc} & \begin{array}{cc} \frac{\partial }{\partial t}\left(u_{0}\left(x,t\right)+w_{0}\left(x,t\right)\right) & =D\;\frac{\partial^{2}}{\partial x^{2}}u_{0}\left(x,t\right)+\kappa_{u}v_{0}\left(x,t\right)-\eta_{b}w_{0}\left(x,t\right)\;,\\ 0 & =\;+\lambda_{b}u_{0}\left(x,t\right)-\lambda_{u}w_{0}\left(x,t\right)\;,\\ \frac{\partial }{\partial t}v_{0}\left(x,t\right) & =\;-\kappa_{u}v_{0}\left(x,t\right)+\eta_{b}w_{0}\left(x,t\right)\;. \end{array} \end{array} \end{array}$$ (17)

From the second equation in (17), we obtain the quasi-steady state relation *w*_0_(*x*, *t*) = *γu*_0_(*x*, *t*), where $\gamma =\frac{\lambda_{b}}{\lambda_{u}}=\frac{\gamma_{b}}{\gamma_{u}}$. If we define *c*_0_(*x*, *t*) = *u*_0_(*x*, *t*) + *w*_0_(*x*, *t*) and use the quasi-steady state, we note that $w_{0}\left(x,t\right)=\frac{\gamma }{1+\gamma }\;c_{0}$ and $u_{0}\left(x,t\right)=\frac{1}{1+\gamma }\;c_{0}$. Substituting this into the first equation, the leading-order system (17) becomes the reaction-diffusion system of two equations

$$\begin{array}{c} \begin{array}{cc} & \begin{array}{cc} \frac{\partial }{\partial t}c_{0}\left(x,t\right) & =\frac{D}{1+\gamma }\;\frac{\partial^{2}}{\partial x^{2}}c_{0}\left(x,t\right)+\kappa_{u}v_{0}\left(x,t\right)-\eta_{b}\frac{\gamma }{1+\gamma }c_{0}\left(x,t\right)\;,\\ \frac{\partial }{\partial t}v_{0}\left(x,t\right) & =\;-\kappa_{u}v_{0}\left(x,t\right)+\eta_{b}\frac{\gamma }{1+\gamma }c_{0}\left(x,t\right)\;. \end{array} \end{array} \end{array}$$ (18)

Thus, we conclude that if the turnover of weakly bound biomolecules to/from a freely diffusing state is sufficiently fast (in other words, if the assumption of high turnover rates *γ*_*b*_ and *γ*_*u*_ is met) the reaction-diffusion system of three Eq (14) can be approximated with the reaction-diffusion system of two Eq (18). An analogous result is obtained for models 6, 7 and 8 (see S3 File in the Supporting Information for details).

## Supporting information

S1 File Source code for model fitting and selection methods written in R. (ZIP)

S2 File FRAP data for histone H1 variants H1.0-H1.5. (ZIP)

S3 File Perturbation analysis on models 6, 7, and 8. (PDF)
