# Supplementary material for: Using a model comparison approach to describe the assembly pathway for histone H1
Source: PLoS One. 2018 Jan 19;13(1):e0191562. doi: 10.1371/journal.pone.0191562 (PMC5774818; doi:10.1371/journal.pone.0191562)
Supplement: S3 File — (PDF) [file pone.0191562.s003.pdf]

S3 File: Perturbation analysis for models 6, 7, and 8

Two-population model

The system of reaction-diffusion equations describing the two population model (model 0) is given by

$$\begin{aligned}\frac{\partial}{\partial t}u(x,t) &= D_{\text{eff}}\,\frac{\partial^2}{\partial x^2}u(x,t) - \kappa_{\text{on}}u(x,t) + \kappa_{\text{off}}v(x,t)\ ,\\ \frac{\partial}{\partial t}v(x,t) &= \qquad\qquad\qquad + \kappa_{\text{on}}u(x,t) - \kappa_{\text{off}}v(x,t)\ ,\end{aligned} \tag{1}$$

where D_{eff} is the effective diffusion coefficient, and k_{on} and k_{off} are the binding and unbinding rates, respectively.

Model 6

The system of reaction-diffusion equations describing model 6 is given by

$$\begin{aligned}\frac{\partial}{\partial t}u(x,t) &= D\,\frac{\partial^2}{\partial x^2}u(x,t) - \gamma_b u(x,t) + \gamma_u w(x,t)\ ,\\ \frac{\partial}{\partial t}w(x,t) &= \qquad - \eta_b w(x,t) + \eta_u v(x,t) + \gamma_b u(x,t) - \gamma_u w(x,t)\ ,\\ \frac{\partial}{\partial t}v(x,t) &= \qquad + \eta_b w(x,t) - \eta_u v(x,t)\ .\end{aligned} \tag{2}$$

Assuming that $\gamma_b = \frac{\lambda_b}{\varepsilon}$, and $\gamma_u = \frac{\lambda_u}{\varepsilon}$, where $\varepsilon << 1$, the reaction-diffusion system (2) can be rewritten as

$$\begin{aligned}\frac{\partial}{\partial t}(u(x,t) + w(x,t)) &= D\,\frac{\partial^2}{\partial x^2}u(x,t) - \eta_b w(x,t) + \eta_u v(x,t)\ ,\\ \epsilon\frac{\partial}{\partial t}w(x,t) &= \qquad - \epsilon\eta_b w(x,t) + \epsilon\eta_u v(x,t) + \lambda_b u(x,t) - \lambda_u w(x,t)\ ,\\ \frac{\partial}{\partial t}v(x,t) &= \qquad + \eta_b w(x,t) - \eta_u v(x,t)\ .\end{aligned} \tag{3}$$

If we now consider a perturbation expansion for $u(x,t)$, $w(x,t)$ and $v(x,t)$ of the form

$$u(x,t) \sim \sum_{n=0}^{\infty} \varepsilon^n u_n(x,t)\ , \quad w(x,t) \sim \sum_{n=0}^{\infty} \varepsilon^n w_n(x,t)\ , \quad v(x,t) \sim \sum_{n=0}^{\infty} \varepsilon^n v_n(x,t)\ , \tag{4}$$

we obtain the following leading-order system for (3)

$$\begin{aligned}\frac{\partial}{\partial t}(u_0(x,t) + w_0(x,t)) &= D\,\frac{\partial^2}{\partial x^2}u_0(x,t) - \eta_b w_0(x,t) + \eta_u v_0(x,t)\ ,\\ 0 &= \qquad + \lambda_b u_0(x,t) - \lambda_u w_0(x,t)\ ,\\ \frac{\partial}{\partial t}v_0(x,t) &= \qquad + \eta_b w_0(x,t) - \eta_u v_0(x,t)\ .\end{aligned} \tag{5}$$

From the second equation in (5), we obtain the quasi-steady state relation $w_0(x,t) = \gamma u_0(x,t)$, where $\gamma = \frac{\lambda_b}{\lambda_u} = \frac{\gamma_b}{\gamma_u}$. If we define $c_0(x,t) = u_0(x,t) + w_0(x,t)$, and use the quasi-steady state, we note that $w_0(x,t) = \frac{\gamma}{1+\gamma}\,c_0$ and $u_0(x,t) = \frac{1}{1+\gamma}\,c_0$. Substituting this into the first equation, the leading-order system (5) becomes the reaction-diffusion system of two equations

$$\begin{aligned}\frac{\partial}{\partial t}c_0(x,t) &= \frac{D}{1+\gamma}\,\frac{\partial^2}{\partial x^2}c_0(x,t) - \frac{\eta_b\gamma}{1+\gamma}c_0(x,t) + \eta_u v_0(x,t)\ ,\\ \frac{\partial}{\partial t}v_0(x,t) &= \qquad + \frac{\kappa_b\gamma}{1+\gamma}c_0(x,t) - \eta_u v_0(x,t)\ .\end{aligned} \tag{6}$$

This is equivalent to (1) with

$$D_{\text{eff}} = \frac{D}{1+\gamma}, \quad \kappa_{\text{on}} = \frac{\eta_b \gamma}{1+\gamma}, \quad \kappa_{\text{off}} = \eta_u.$$

Thus, we conclude that if the turnover of weakly bound biomolecules to/from a freely diffusing state is sufficiently fast the reaction-diffusion system of three equations (2) can be approximated with the reaction-diffusion system of two equations (6).

If we use the solution of the reaction-diffusion system of equations (2) to fit histone H1.5 data assuming a diffusion coefficient $D = 25\,\mu\text{m}^2/\text{s}$, we obtain parameter values of

$$\gamma_b = 0.099881, \quad \gamma_u = 0.014805, \quad \eta_b = 0.001161, \quad \eta_u = 0.002231. \tag{7}$$

If we use those values to estimate parameters in (1), we obtain

$$D_{\text{eff}} = 3.227462, \quad \kappa_{\text{on}} = 0.001011, \quad \kappa_{\text{off}} = 0.002231. \tag{8}$$

In Fig. 1 (left panel) we show the recovery curve according to both model 6 (2) with parameter values (7), and its leading-order approximation (6) equivalent to the two population model (1) with parameter values (8).

Alternativetly, we could fit first the leading order approximation (6), and then estimate parameters in model 6 (5) according to the relation

$$\gamma = \frac{D}{D_{\text{eff}}} - 1, \quad \eta_b = \kappa_{\text{on}} \frac{1+\gamma}{\gamma}, \quad \eta_u = \kappa_{\text{off}}.$$

However, parameters γ_b and γ_u cannot be uniquely determined. By fitting directly the leading order approximation (6) to the same histone H1.5 data, we obtain the following parameter estimates

$$D_{\text{eff}} = 0.0047682216, \quad \kappa_{\text{on}} = 0.0002122792, \quad \kappa_{\text{off}} = 0.0011231743. \tag{9}$$

Assuming a diffusion coefficient $D = 25\,\mu\text{m}^2/\text{s}$, we can estimate the following parameters for model 6

$$\gamma = 5242.045, \quad \eta_b = 0.00021, \quad \eta_u = 0.00112. \tag{10}$$

In Fig. 1 (right panel), we used these parameter estimates to sketch the explicit solution of the full model 6 for different values of γ_u and γ_b, keeping their ratio γ fixed.

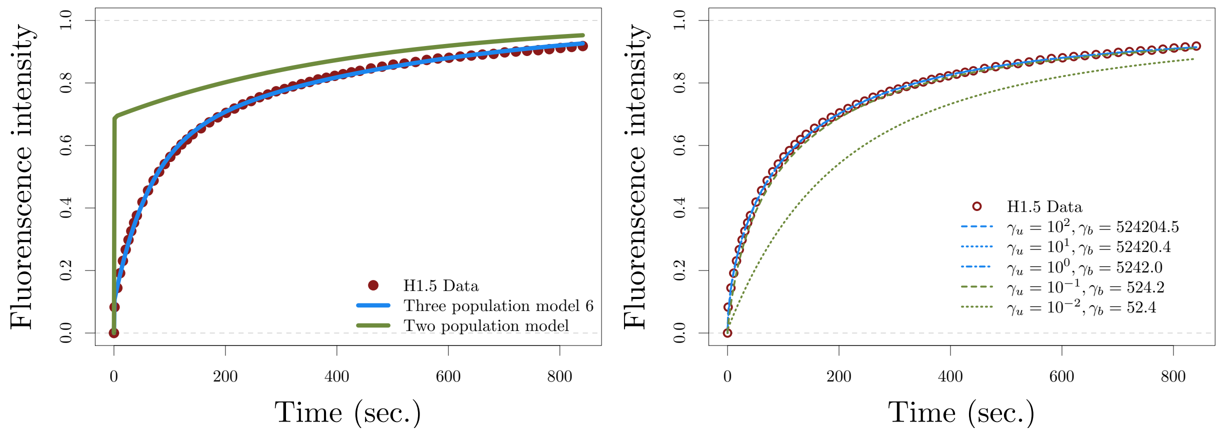

Figure 1: Left panel: Fitting of the three-population model 6 (blue) and its two population leading-order approximation (green) to histone H1.5 data. Right panel: Recovery curves of the three population model 6 approximating H1.5 data for several parameter estimates with values greater than two orders of magnitude.

Model 7

The system of reaction-diffusion equations describing model 7 is given by

$$\begin{aligned}
\frac{\partial}{\partial t}u(x,t) &= D\,\frac{\partial^2}{\partial x^2}u(x,t) - \gamma_b u(x,t) + \gamma_u w(x,t) - \kappa_b u(x,t) + \kappa_u v(x,t)\ ,\\
\frac{\partial}{\partial t}w(x,t) &= \qquad\qquad + \gamma_b u(x,t) - \gamma_u w(x,t)\ ,\\
\frac{\partial}{\partial t}v(x,t) &= \qquad\qquad + \kappa_b u(x,t) - \kappa_u v(x,t)\ .
\end{aligned} \tag{11}$$

Assuming that $\gamma_b = \frac{\lambda_b}{\varepsilon}$, and $\gamma_u = \frac{\lambda_u}{\varepsilon}$, where $\varepsilon << 1$, the reaction-diffusion system (11) can be rewritten as

$$\begin{aligned}
\frac{\partial}{\partial t}(u(x,t) + w(x,t)) &= D\,\frac{\partial^2}{\partial x^2}u(x,t) - \kappa_b u(x,t) + \kappa_u v(x,t)\ ,\\
\epsilon\frac{\partial}{\partial t}w(x,t) &= \qquad\qquad + \lambda_b u(x,t) - \lambda_u w(x,t)\ ,\\
\frac{\partial}{\partial t}v(x,t) &= \qquad\qquad + \kappa_b u(x,t) - \kappa_u v(x,t)\ .
\end{aligned} \tag{12}$$

If we now consider a perturbation expansion for $u(x,t)$, $w(x,t)$ and $v(x,t)$ of the form

$$u(x,t) \sim \sum_{n=0}^{\infty} \varepsilon^n u_n(x,t)\ , \quad w(x,t) \sim \sum_{n=0}^{\infty} \varepsilon^n w_n(x,t)\ , \quad v(x,t) \sim \sum_{n=0}^{\infty} \varepsilon^n v_n(x,t)\ , \tag{13}$$

we obtain the following leading-order system for (12)

$$\begin{aligned}
\frac{\partial}{\partial t}(u_0(x,t) + w_0(x,t)) &= D\,\frac{\partial^2}{\partial x^2}u_0(x,t) - \kappa_b u_0(x,t) + \kappa_u v_0(x,t)\ ,\\
0 &= \qquad\qquad + \lambda_b u_0(x,t) - \lambda_u w_0(x,t)\ ,\\
\frac{\partial}{\partial t}v_0(x,t) &= \qquad\qquad + \kappa_b u_0(x,t) - \kappa_u v_0(x,t)\ .
\end{aligned} \tag{14}$$

From the second equation in (14), we obtain the quasi-steady state relation $w_0(x,t) = \gamma u_0(x,t)$, where $\gamma = \frac{\lambda_b}{\lambda_u} = \frac{\gamma_b}{\gamma_u}$. If we define $c_0(x,t) = u_0(x,t) + w_0(x,t)$, and use the quasi-steady state, we note that $w_0(x,t) = \frac{\gamma}{1+\gamma}\,c_0$ and $u_0(x,t) = \frac{1}{1+\gamma}\,c_0$. Substituting this into the first equation, the leading-order system (14) becomes the reaction-diffusion system of two equations

$$\begin{aligned}
\frac{\partial}{\partial t}c_0(x,t) &= \frac{D}{1+\gamma}\,\frac{\partial^2}{\partial x^2}c_0(x,t) - \frac{\kappa_b}{1+\gamma}c_0(x,t) + \kappa_u v_0(x,t)\ ,\\
\frac{\partial}{\partial t}v_0(x,t) &= \qquad\qquad + \frac{\kappa_b}{1+\gamma}c_0(x,t) - \kappa_u v_0(x,t)\ .
\end{aligned} \tag{15}$$

This is equivalent to (1) with

$$D_{\text{eff}} = \frac{D}{1+\gamma}, \quad \kappa_{\text{on}} = \frac{\kappa_b}{1+\gamma}, \quad \kappa_{\text{off}} = \kappa_u.$$

Thus, we conclude that if the turnover of weakly bound biomolecules to/from a freely diffusing state is sufficiently fast the reaction-diffusion system of three equations (11) can be approximated with the reaction-diffusion system of two equations (15).

If we use the solution of the reaction-diffusion system of equations (11) to fit histone H1.5 data assuming a diffusion coefficient $D = 25\,\mu\text{m}^2/\text{s}$, we obtain parameter values of

$$\kappa_b = 0.009542, \quad \kappa_u = 0.002045, \quad \gamma_b = 0.090338, \quad \gamma_u = 0.016153 \tag{16}$$

If we use those values to estimate parameters in (1), we obtain

$$D_{\text{eff}} = 3.792223, \quad \kappa_{\text{on}} = 0.001447, \quad \kappa_{\text{off}} = 0.002045. \tag{17}$$

In Fig. 2 (left panel) we show the recovery curve according to both model 7 (11) with parameter values (16), and its leading-order approximation (15) equivalent to the two population model (1) with parameter values (17).

Alternativetly, we could fit first the leading order approximation (15), and then estimate parameters in model 7 (14) according to the relation

$$\gamma = \frac{D}{D_{\text{eff}}} - 1, \quad \kappa_b = \kappa_{\text{on}}(1+\gamma), \quad \kappa_u = \kappa_{\text{off}}.$$

However, parameters γ_b and γ_u cannot be uniquely determined. By fitting directly the leading order approximation (15) to the same histone H1.5 data, we obtain the following parameter estimates

$$D_{\text{eff}} = 0.0047682216, \quad \kappa_{\text{on}} = 0.0002122792, \quad \kappa_{\text{off}} = 0.0011231743. \tag{18}$$

Assuming a diffusion coefficient $D = 25\,\mu\text{m}^2/\text{s}$, we can estimate the following parameters for model 7

$$\gamma = 5242.045, \quad \kappa_b = 1.11299, \quad \kappa_u = 0.00112. \tag{19}$$

In Fig. 2 (right panel), we used these parameter estimates to sketch the explicit solution of the full model 6 for different values of γ_u and γ_b, keeping their ratio γ fixed.

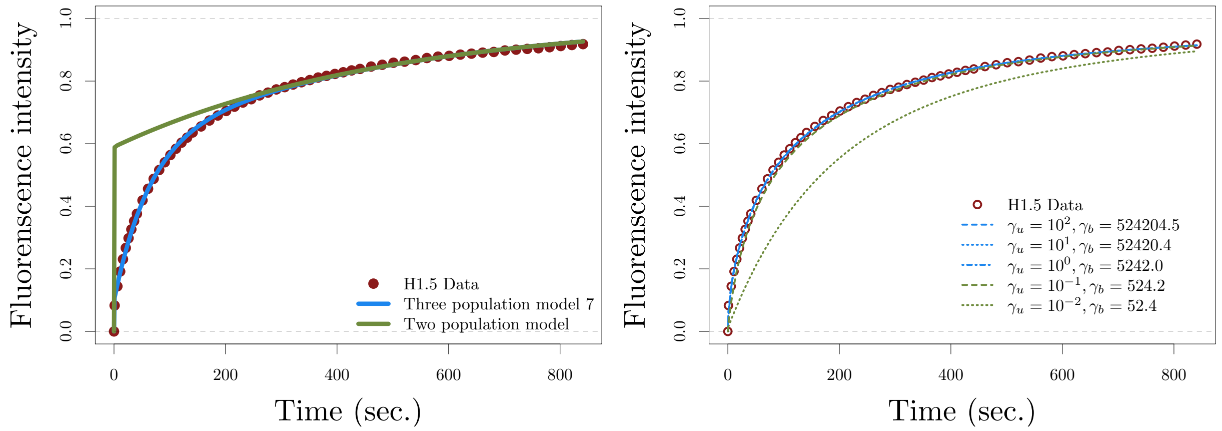

Figure 2: Left panel: Fitting of the three-population model 7 (blue) and its two population leading-order approximation (green) to histone H1.5 data. Right panel: Recovery curves of the three population model 7 approximating H1.5 data for several parameter estimates with values greater than two orders of magnitude.

Model 8

The system of reaction-diffusion equations describing model 8 is given by

$$\begin{aligned}
\frac{\partial}{\partial t}u(x,t) &= D\,\frac{\partial^2}{\partial x^2}u(x,t) - \gamma_b u(x,t) + \gamma_u w(x,t) - \kappa_b u(x,t)\ , \\
\frac{\partial}{\partial t}w(x,t) &= \qquad\qquad\quad + \eta_u v(x,t) + \gamma_b u(x,t) - \gamma_u w(x,t)\ , \\
\frac{\partial}{\partial t}v(x,t) &= \qquad\qquad\quad + \kappa_b u(x,t) - \eta_u v(x,t)\ .
\end{aligned} \tag{20}$$

Assuming that $\gamma_b = \frac{\lambda_b}{\varepsilon}$, and $\gamma_u = \frac{\lambda_u}{\varepsilon}$, where $\varepsilon << 1$, the reaction-diffusion system (20) can be rewritten as

$$\begin{aligned}
\frac{\partial}{\partial t}(u(x,t)+w(x,t)) &= D\,\frac{\partial^2}{\partial x^2}u(x,t) - \kappa_b u(x,t) + \eta_u v(x,t)\ ,\\
\epsilon\frac{\partial}{\partial t}w(x,t) &= \qquad\qquad + \epsilon\eta_u v(x,t) + \lambda_b u(x,t) - \lambda_u w(x,t)\ ,\\
\frac{\partial}{\partial t}v(x,t) &= \qquad\qquad + \kappa_b u(x,t) - \eta_u v(x,t)\ .
\end{aligned} \tag{21}$$

If we now consider a perturbation expansion for $u(x,t)$, $w(x,t)$ and $v(x,t)$ of the form

$$u(x,t) \sim \sum_{n=0}^{\infty} \varepsilon^n u_n(x,t)\ , \quad w(x,t) \sim \sum_{n=0}^{\infty} \varepsilon^n w_n(x,t)\ , \quad v(x,t) \sim \sum_{n=0}^{\infty} \varepsilon^n v_n(x,t)\ , \tag{22}$$

we obtain the following leading-order system for (21)

$$\begin{aligned}
\frac{\partial}{\partial t}(u_0(x,t)+w_0(x,t)) &= D\,\frac{\partial^2}{\partial x^2}u_0(x,t) - \kappa_b u_0(x,t) + \eta_u v_0(x,t)\ ,\\
0 &= \qquad\qquad + \lambda_b u_0(x,t) - \lambda_u w_0(x,t)\ ,\\
\frac{\partial}{\partial t}v_0(x,t) &= \qquad\qquad + \kappa_b u_0(x,t) - \eta_u v_0(x,t)\ .
\end{aligned} \tag{23}$$

From the second equation in (23), we obtain the quasi-steady state relation $w_0(x,t) = \gamma u_0(x,t)$, where $\gamma = \frac{\lambda_b}{\lambda_u} = \frac{\gamma_b}{\gamma_u}$. If we define $c_0(x,t) = u_0(x,t) + w_0(x,t)$, and use the quasi-steady state, we note that $w_0(x,t) = \frac{\gamma}{1+\gamma}\,c_0$ and $u_0(x,t) = \frac{1}{1+\gamma}\,c_0$. Substituting this into the first equation, the leading-order system (23) becomes the reaction-diffusion system of two equations

$$\begin{aligned}
\frac{\partial}{\partial t}c_0(x,t) &= \frac{D}{1+\gamma}\,\frac{\partial^2}{\partial x^2}c_0(x,t) - \frac{\kappa_b}{1+\gamma}c_0(x,t) + \eta_u v_0(x,t)\ ,\\
\frac{\partial}{\partial t}v_0(x,t) &= \qquad\qquad + \frac{\kappa_b}{1+\gamma}c_0(x,t) - \eta_u v_0(x,t)\ .
\end{aligned} \tag{24}$$

This is equivalent to (1) with

$$D_{\text{eff}} = \frac{D}{1+\gamma}, \quad \kappa_{\text{on}} = \frac{\kappa_b}{1+\gamma}, \quad \kappa_{\text{off}} = \eta_u.$$

Thus, we conclude that if the turnover of weakly bound biomolecules to/from a freely diffusing state is sufficiently fast the reaction-diffusion system of three equations (20) can be approximated with the reaction-diffusion system of two equations (24).

If we use the solution of the reaction-diffusion system of equations (20) to fit histone H1.5 data assuming a diffusion coefficient $D = 25\,\mu\text{m}^2/\text{s}$, we obtain parameter values of

$$\kappa_b = 0.008333, \quad \gamma_b = 0.091547, \quad \gamma_u = 0.016153, \quad \eta_u = 0.002045. \tag{25}$$

If we use those values to estimate parameters in (1), we obtain

$$D_{\text{eff}} = 3.749684, \quad \kappa_{\text{on}} = 0.001249, \quad \kappa_{\text{off}} = 0.002045. \tag{26}$$

In Fig. 3 (left panel) we show the recovery curve according to both model 8 (20) with parameter values (25), and its leading-order approximation (24) equivalent to the two population model (1) with parameter values (26).

Alternativetly, we could fit first the leading order approximation (24), and then estimate parameters in model 8 (23) according to the relation

$$\gamma = \frac{D}{D_{\text{eff}}} - 1, \quad \kappa_b = \kappa_{\text{on}}(1+\gamma), \quad \eta_u = \kappa_{\text{off}}.$$

However, parameters γ_b and γ_u cannot be uniquely determined. By fitting directly the leading order approximation (24) to the same histone H1.5 data, we obtain the following parameter estimates

$$D_{\text{eff}} = 0.0047682216, \quad \kappa_{\text{on}} = 0.0002122792, \quad \kappa_{\text{off}} = 0.0011231743. \tag{27}$$

Assuming a diffusion coefficient $D = 25\,\mu\text{m}^2/\text{s}$, we can estimate the following parameters for model 8

$$\gamma = 5242.045, \quad \kappa_b = 1.11299, \quad \eta_u = 0.00112. \tag{28}$$

In Fig. 3 (right panel), we used these parameter estimates to sketch the explicit solution of the full model 8 for different values of γ_u and γ_b, keeping their ratio γ fixed.

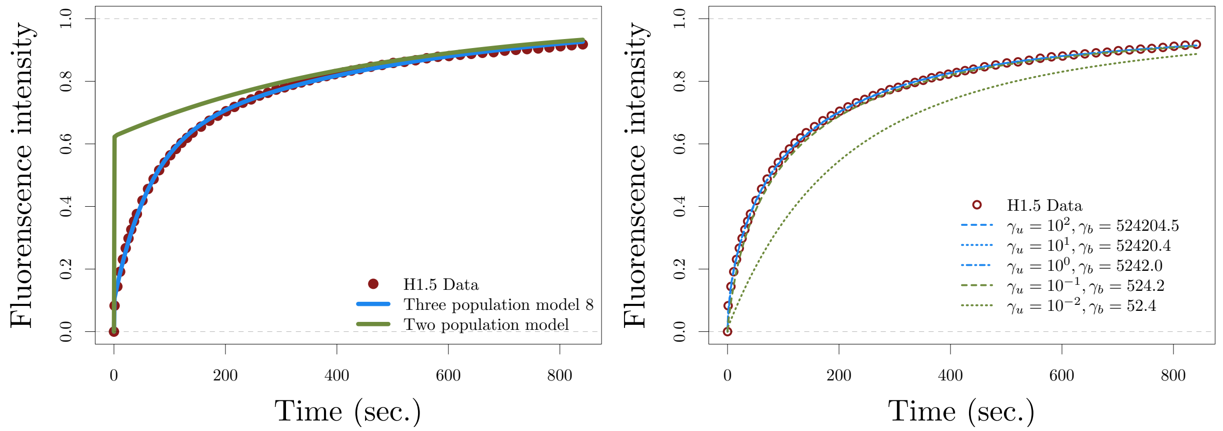

Figure 3: Left panel: Fitting of the three-population model 8 (blue) and its two population leading-order approximation (green) to histone H1.5 data. Right panel: Recovery curves of the three population model 8 approximating H1.5 data for several parameter estimates with values greater than two orders of magnitude.

Remarks

As explained in the manuscript, Figs. 1–3 show that the recovery curve obtained from fitting any of the three-population models is significantly different from their leading-order approximations for parameter estimates not greater than two orders of magnitude, but equivalent for parameter estimates greater two orders of magnitude. This occurs because the turnover of weakly bound biomolecules to/from a freely diffusing state is not sufficiently fast when the rapid interaction is characterized with parameters values not greater than two orders of magnitude; in other words, the assumption of high turnover rates (γ_b and γ_u) is not met.
